# Kidney Injury Molecule-1 and Cardiovascular Diseases: From Basic Science to Clinical Practice

**DOI:** 10.1155/2015/854070

**Published:** 2015-11-30

**Authors:** Branislava Medić, Branislav Rovčanin, Gordana Basta Jovanović, Sanja Radojević-Škodrić, Milica Prostran

**Affiliations:** ^1^Department of Pharmacology, Clinical Pharmacology and Toxicology, Faculty of Medicine, University of Belgrade, Dr Subotica Starijeg 1, 11129 Belgrade, Serbia; ^2^Center for Endocrine Surgery, Clinical Center of Serbia, Faculty of Medicine, University of Belgrade, Koste Todorovica 8, 11000 Belgrade, Serbia; ^3^Department of Pathology, Faculty of Medicine, University of Belgrade, Dr Subotica Starijeg 1, 11129 Belgrade, Serbia

## Abstract

Despite the recent findings concerning pathogenesis and novel therapeutic strategies, cardiovascular disease (CVD) still stays the leading cause of morbidity and mortality in patients with renal dysfunction, especially acute kidney injury (AKI). Early detection of patients with impaired renal function with cardiovascular risk may help ensure more aggressive treatment and improve clinical outcome. Kidney injury molecule-1 (KIM-1) is a new, promising marker of kidney damage which is currently the focus of countless studies worldwide. Some recent animal and human studies established KIM-1 as an important marker of acute tubular necrosis (ATN) and reliable predictor of development and prognosis of AKI. Food and Drug Administration (FDA) in USA acclaimed KIM-1 as an AKI biomarker for preclinical drug development. Recent data suggest the importance of monitoring of KIM-1 for early diagnosis and clinical course not only in patients with various forms of AKI and other renal diseases but also in patients with cardiorenal syndrome, heart failure, cardiopulmonary bypass, cardiothoracic surgical interventions in the pediatric emergency setting, and so forth. The aim of this review article is to summarize the literature data concerning KIM-1 as a potential novel marker in the early diagnosis and prediction of clinical outcome of certain cardiovascular diseases.

## 1. Background

**Figure 1:**
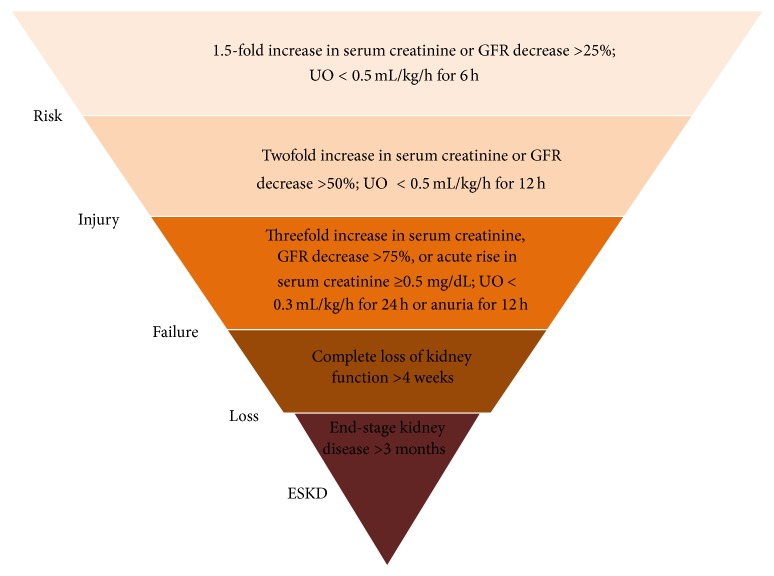
RIFLE classification [Risk-Injury-Failure-Loss-End-stage kidney disease (ESKD)]; GFR, glomerular filtration rate; UO, urine output (modified by [1]).

Despite the recent findings concerning pathogenesis and novel therapeutic strategies, cardiovascular disease (CVD) still stays the leading cause of morbidity and mortality in patients with renal dysfunction, especially acute kidney injury (AKI). The term “acute kidney injury” (AKI) represents a wide range of structural and functional renal changes from mild alteration to complete organ failure [1]. RIFLE classification [Risk-Injury-Failure-Loss-End-stage kidney disease (ESKD)] was established by the Acute Dialysis Quality Initiative (ADQI) group in order to supply the obligation for uniform definition, early detection, and grading of AKI. After this, the AKIN criteria were created by the Acute Kidney Injury Network (AKIN) for further refinement of the definition of AKI [1]. The RIFLE classification criteria are shown in Figure 1.

In spite of advances in the understanding of pathogenesis of AKI and progress in classifying different forms depending on the etiology, clinic manifestations, and stages as well as the novel therapeutic strategies, the mortality rate still remains high (approximately 2 million people worldwide pass away because of AKI and its consequences every year) [2–4]. One of the principal reasons for this poor prognosis is too late detection of renal impairment and the preventive strategies are most effective when they are started before oliguria. Serum creatinine is still gold standard of kidney injury although it is well known as an insensitive and unreliable biomarker (e.g., its concentration does not increase significantly until about half of the kidney function is lost) [5, 6].

Considering these data, scientists and clinicians worldwide were making great efforts in the past decade in order to discover and validate novel AKI biomarkers. The search for such biomarker can be specified as “the search for renal troponin I” [7]. The term biomarker (acronym for biological marker) is used to define a characteristic that can be measured and evaluated as normal biological process, pathological process, or pharmacological response to therapeutic intervention [8, 9].

Numerous studies and the previous experience have shown that the ideal marker for AKI should be (1) noninvasive, (2) easily detectable in accessible body samples (e.g., serum or urine), (3) highly sensitive and specific for AKI, (4) rapidly and reliably measurable, (5) capable of early detection, (6) predictor of AKI severity and prognosis, (7) unaffected by other biological variables, (8) inexpensive, and so forth [10].

KIM-1 is one of the most promising, early biomarkers due to its translatability between preclinical and clinical trials. It is believed that this molecule participates in the process of both kidney injury and healing, although precise mechanism of restoration of tubular integrity after injury still remains unclear. In the past 15 years Ichimura and collaborators have published several papers regarding importance and clinical applicability of new biomarkers of acute renal failure [11–15]. Recently, his team has demonstrated that in ischemic injury KIM-1 expression is most prominent in S3 segment (i.e., the segment most susceptible to ischemic injury) [11]. Numerous animal and human studies recognized KIM-1 as an early and reliable predictor of AKI [12, 16, 17].

The aim of this review article is to summarize and discuss the literature data concerning KIM-1 as a potential novel marker in the early diagnosis and prediction of clinical outcome of certain cardiovascular diseases.

## 2. KIM-1: Molecular Structure

Kim-1 protein is a membrane receptor for human hepatitis A virus (HHAV) and T-cell immunoglobulin and mucin domain containing 4 (TIMD 4). KIM-1 is a single pass type I cell membrane glycoprotein which contains, in its extracellular section, a six-cysteine immunoglobulin-like domain, two N-glycosylation sites, and T/SP rich domain characteristic of mucin-like O-glycosylated proteins. Kim-1 has one transmembrane domain and a short intracellular domain which contains a signaling motif for tyrosine phosphorylation present in the renal form of protein (Kim-1b). The structure of the protein led to the conclusion that it has adhesion properties, but, later on, its diverse biological functions were revealed [18].

Kim-1 gene is located in chromosome 5q33.2. It is widely expressed with highest levels in kidney and testis. It is also expressed by activated CD4+ T-cells during the development of helper T-cell response [21]. The gene is upregulated in the kidney in renal diseases, which was confirmed at the protein level [22]. The reference genome represents an allele that retains MTTVP amino acid (allelic variant with the 5-amino acid insertion at position 158) segment that confers protection against atopy in HHAV seropositive individuals. Alternative splicing of this gene results in multiple transcript variants. The related pseudogenes have been identified on chromosomes 4, 12, and 19 [23].

Among mammals the gene for Kim-1 is highly conserved, suggesting its biological importance and low evolutionary plasticity, particularly of its extracellular and intracellular domains, with the conservation score 921. The amino acid sequence of Kim-1 genes, belonging to the* Homo sapiens*,* Rattus norvegicus*,* Mus musculus*, and* Felis catus*, was obtained from the UniProtKB/Swiss-Prot database and aligned by T-COFFEE (tree-based consistency objective function for alignment evaluation) bioinformatic tool [24].

Similarity between human extracellular domain and rat extracellular domain of Kim-1 protein was determined by comparison of amino acid sequences. Using the UniProt database, amino acid sequences of human and rat Kim-1 were accessed by following entries Q96D42 and O54947, respectively. Sequence alignment was performed by using BLAST (Basic Local Alignment Search Tool) [25].

**Figure 2:**
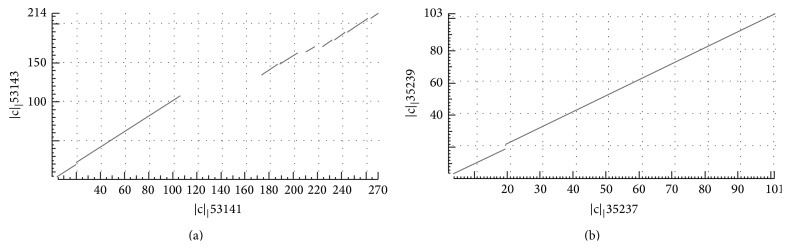
Dot matrix view of human EC Kim-1 domain and rat's EC Kim-1 domain and Ig like V subdomain aligned in BLAST. (a) Human sequence is plotted on *x*-axis and rats sequence is plotted on *y*-axis. Several gaps demonstrate the existence of repeated amino acid sequences, which exist only in human ortholog of Kim-1. (b) Human sequence is plotted on *x*-axis and rats sequence is plotted on *y*-axis. Single gap shows the two amino acids which are present only in rat's ortholog.

These domains show 57% of sequence identity (*E*-value 5*e* − 41) and most of differences are due to the existence of repeated elements in human EC domain of Kim-1. The aminoacid sequence similarity between human's and rat's Ig like V subdomain of EC domain is 57% (*E*-value 2*e* − 39). Similarity between human and rat Kim-1 EC domain suggests that structural conditions are fulfilled for equalization of ligand binding and overall biochemical similarity. The sequence conservation in both species suggests the significant selective pressure against sequence alteration during evolution of Kim-1 gene. The human anti-EC Kim-1 Mab binds to the same domain of rat's Kim-1, leading to a conclusion that experimental results based on rat's model could be seriously taken into concern for extrapolation to humans [26]. Dot matrix view of human EC Kim-1 domain and rat's EC Kim-1 domain and Ig like V subdomain aligned in BLAST program is shown in Figure 2.

Rat and human cDNAs encoding KIM-1 (KIM-1 in the rat) were identified for the first time using difference analysis between normal kidneys and kidneys exposed to ischemia/reperfusion (I/R) injury followed by regeneration of proximal tubular cells. KIM-1 is found to be expressed at low to undetectable levels in the normal adult rat kidney but is markedly expressed by the epithelial proximal tubular cells in response to ischemic or toxic AKI [13, 18]. Modern molecular cytogenetic techniques (*in situ* hybridization and immunohistochemistry) indicated KIM-1 as a marker of proliferation and regeneration in proximal tubules [14].

Some later reports showed mechanism of dropping of KIM-1 ectodomain cells into the urine after proximal tubular injury* in vivo* in rats and rodents [11, 27–29].

Besides that, after injury, KIM-1 acts as a phosphatidylserine receptor that shows the ability to recognize and phagocytose dead cells presented in the postischemic kidney [15, 23].

## 3. KIM-1 in Preclinical Studies

Since the identification of KIM-1 upregulation in the rat model of renal ischemia, additional studies have been performed in order to examine the diagnostic role of KIM-1 in other models of AKI. Vaidya et al. stressed out the importance and practical application of determination of KIM-1 concentration in urine in these kinds of experiments [12]. Ichimura and colleagues examined tissue and urinary KIM-1 expression in a cisplatin-induced nephrotoxicity in rats and proved KIM-1 as a faster and superior marker compared to serum creatinine. A further advance was the development of a sensitive microbead-based KIM-1 ELISA in order to confirm and facilitate the use of urinary KIM-1 as a biomarker of AKI in animal studies [18, 30, 31].

At this moment it is possible to measure KIM-1 concentration in tissue samples and urine and plasma/serum in a simple, rapid, and accurate manner. Some recent studies confirmed KIM-1 as an important biomarker of AKI and acute tubular necrosis (ATN) and showed correlation between its concentration and the degree of renal dysfunction. Renal and urinary Kim-1 correlated with proteinuria and interstitial damage [32].

Adjusted for age, gender, and length of time delay between insult and sampling, a one-unit increase in normalized KIM-1 was associated with a greater than 12-fold increase in the presence of acute tubular necrosis (ATN) [16].

In a recent preclinical study, the diagnostic value of urinary KIM-1 significantly exceeded traditional biomarkers (serum creatinine and urea) as predictors of kidney tubular histopathological changes in rats [33].

Recently, research group from our project provided the first evidence that KIM-1 staining scores could be used as an indicator of the therapeutic benefit of different pharmacological agents in the experimental model of renal ischemia/reperfusion (I/R) injury. KIM-1 reliably confirmed that chloroquine affords an acute protective effect on kidney function and morphology [26].

**Figure 3:**
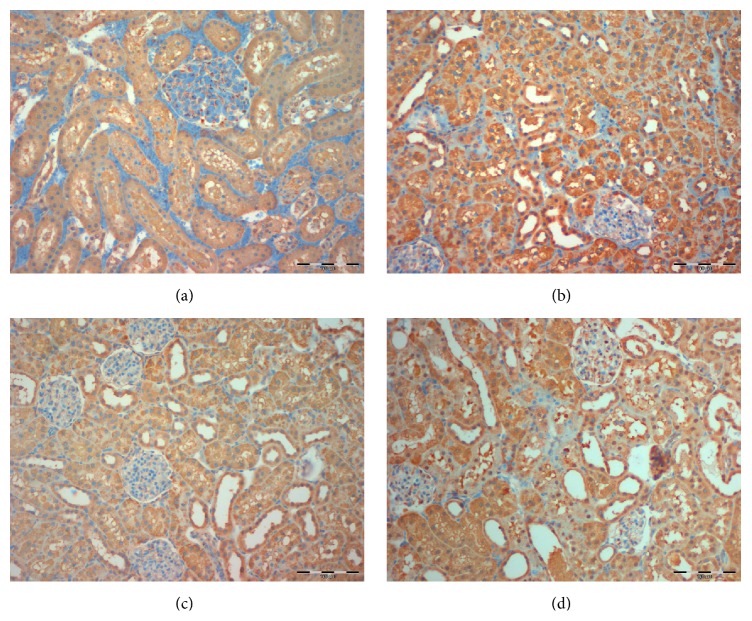
The effects of chloroquine (0.3 mg/kg, i.v; 3 mg/kg, i.v.) on renal I/R injury and histological micrographs of renal tissues: KIM-1 staining score. Chloroquine, in dose of 0.3 and 3 mg/kg, i.v., was injected 30 min before ischemia. Control groups, Sham + Saline, and IR + Saline received instead of drug i.v. bolus of 0.5 mL saline only (unpublished data from our laboratory projects: Professor Milica Prostran (ON175023) together with Professor Gordana Basta-Jovanovic (ON175059)). Histological micrographs of renal tissues: kidney sections taken from Sham-operated rats or rats subjected to renal I/R injury. Kidney injury molecule-1 (KIM-1) staining. Original magnification ×200. Figures were randomly chosen from the series of at least 6 experiments (a–d). (a) Sham-operated animals treated with saline only: absence of immunoreactivity for KIM-1. (b) Rats subjected to renal I/R injury, pretreated with chloroquine at 0.3 mg/kg, i.v. 30 min, before ischemia: most of proximal and some distal tubules show mild staining for KIM-1. (c) Rats subjected to renal I/R injury, pretreated with chloroquine at 3 mg/kg, i.v. 30 min, before ischemia: most of proximal and some distal tubules show moderate staining for KIM-1. (d) Rats subjected to renal I/R injury, pretreated with saline only: proximal and distal tubules show moderate to intensive positive KIM staining.

Unpublished data from our laboratory project are presented in Figure 3.

In addition, KIM-1 has been approved by the US Food and Drug Administration as an AKI biomarker for preclinical drug development [34].

## 4. KIM-1 in Clinical Studies

Subsequent studies in adults suggested that KIM-1 can discriminate patients with different types of acute tubular necrosis (hospitalized patients, critically ill patients, and patients with acute graft rejection) from those without AKI [22]. In hospitalized patients with established AKI, urinary KIM-1 levels predicted adverse clinical outcomes such as dialysis requirement and mortality [35].

A rapid testing method for KIM-1 has been described, yielding semiquantitative results in just 15 minutes [36, 37]. One prospective study has shown that KIM-1 can even predict adverse clinical outcomes in patients with AKI: patients with the highest levels in urinary KIM-1 had the highest odds for dialysis and hospital death [33, 38, 39].

Additional studies have confirmed that KIM-1 urinary concentration is upregulated in various kidney diseases including diabetic nephropathy, focal glomerulosclerosis, membranoproliferative glomerulonephritis, IgA nephropathy, and even renal cell carcinoma [40].

Recent data suggest the importance of monitoring this marker for early diagnosis, prognosis, and the therapy effects not only in patients with various forms of AKI and other renal diseases but also in patients with heart failure after cardiopulmonary bypass, various forms of cardiorenal syndrome, cardiothoracic surgical interventions in the pediatric emergency setting, and so forth.

In the next paragraph, we will discuss the importance of determining KIM-1 markers in certain clinical entities.

## 5. KIM-1 in Cardiorenal Syndrome

Cardiorenal syndrome (CRS) commonly represents complex interaction between heart and kidneys in which acute or chronic dysfunction in one organ may induce acute or chronic dysfunction in the other organ [41]. The pathophysiology of this clinical entity includes reduced renal perfusion, increased venous pressure, and activation of multiple neurohormonal systems, although whole process is still not completely understood [42, 43].

Five different subtypes of CRS have recently been proposed: type 1, acute cardiorenal syndrome (acute impairment of heart function leads to kidney injury and/or dysfunction), type 2, chronic cardiorenal syndrome (chronic heart diseases lead to kidney injury and/or dysfunction), type 3, acute renocardiac syndrome (acute impairment of kidney function leads to heart injury and/or dysfunction), type 4, chronic renocardiac syndrome (chronic kidney disease leads to heart disease and/or dysfunction), and type 5, secondary CRS occurring in systemic disorders (e.g., sepsis, diabetes mellitus, and amyloidosis) simultaneously causing both cardiac and renal dysfunctions [44–49].

Clinical outcome of this syndrome remains poor with a high mortality rate, partly because of delayed diagnosis (approximately 24 h after the event). For this reason, numerous studies are currently underway to confirm the clinical utility of the new biomarkers. In one of them, Damman et al. recently confirmed KIM-1 as an excellent predictive marker for the detection of acute tubular injury in patients with chronic heart failure (HF) after the suspension and the reintroduction of diuretic therapy. KIM-1 levels increased significantly as early as 8 h after diuretics were stopped, remained elevated within three days, and then returned to normal levels as early as 4 h after furosemide was resumed [50]. In this study KIM-1 defeated other markers (such as NGAL and N-acetyl-*β*-D-glucosaminidase (NAG)) and showed how changes in volume status can lead to subclinical tubular injury that may be undetected by traditional biomarkers.

Also, it is shown that urinary KIM-1 was also associated with increased risk of death or hospitalization, independent of GFR in patients with chronic heart failure [51]. Still, for more precise role of KIM-1 in early detection and/or evaluation of therapy in CRS, it is necessary to conduct more comprehensive evaluation.

## 6. KIM-1 in Cardiac Surgery-Associated AKI

Cardiac surgery-associated acute kidney injury (CSA-AKI) often includes coronary artery bypass grafting (CABG), surgery for valvular disease, and congenital heart surgery reportedly occurring in 30%–40% of cases (according to some authors, CRS could be defined as a particular type of type 1 cardiorenal syndrome for which no clear understanding of pathogenesis exists [52]).

It represents the second most common cause of AKI in the intensive care units and an independent predictor after cardiac surgery [53–55]. Hemodynamic and inflammatory factors that lead to oxidation from reactive oxygen species represent major determinants in poor prognosis of cardiac surgery-associated AKI [56].

Recent studies confirmed that increases in serum creatinine concentration are observed too late (usually within 48 h) and have significant impact on mortality rate in these cases [54, 57, 58]. This finding was one of the reasons to start with examination of novel biomarkers in clinical trials in CSA-AKI.

Some of the first investigations were carried out on patients undergoing CBP where KIM-1 levels increased significantly at both 2 hours and 24 hours after operation in patients with AKI [59, 60]. Similar results were found in a small case-control study of 40 pediatric patients following CPB [61].

Koyner et al. compared the ability of several biomarkers to predict the progression of kidney damage in patients with elevated serum creatinine concentration levels who underwent cardiac surgery. KIM-1 was shown as a predictor of secondary importance in these situations [62]. Conclusion of the research conducted by Hall and colleagues was the fact that urine NGAL had the best results, followed by KIM-1 and IL-18 [63]. Arthur and associates evaluated ability of 32 AKI biomarkers to predict declining of renal function in patients with AKIN stage 1 AKI after cardiac surgery. Although they found IL-18, independently, as a best performer, it was demonstrated that combination of KIM-1 and IL-18 was much more accurate in prediction [64].

Another study measured KIM-1, NAG, and NGAL in 90 adults undergoing cardiac surgery. The values of area under the curve (AUC) in prediction of AKI immediately and 3 h after operation were 0.68 and 0.65 for KIM-1, 0.61 and 0.63 for NAG, and 0.59 and 0.65 for NGAL, respectively. Combining the three biomarkers enhanced the sensitivity of early detection of postoperative AKI compared with individual biomarkers: the AUCs for the three biomarkers combined were 0.75 and 0.78 [65].

Recently, it was shown that preoperative KIM-1 urinary level is able to predict the development of AKI in adults undergoing cardiac surgery [62, 66]. Also, KIM-1 showed potential of being a good predictor of development of AKI in pediatric cardiorenal injuries in emergency settings. For example, Han et al. found that urinary KIM-1 detected AKI before serum creatinine in a cohort of children undergoing cardiopulmonary bypass (CBP) [67]. Krawczeski et al. proved evidence that KIM-1 at 12 h following CPB independently correlated with CPB time and risk adjustment for congenital heart surgery score (RACHS-1) [68].

Contrary to previous research, Hazle et al. did not confirm KIM-1 as a good prognostic factor in children. They measured urinary levels of few novel biomarkers (neutrophil gelatinase-associated lipocalin (NGAL), interleukin-18 (IL-18), kidney injury molecule-1 (KIM-1), and cystatin C) pre- and postoperatively in infants younger than 6 months of age to predict outcomes following congenital heart surgery. It was shown that KIM-1 poorly differentiated patients with either good or poor outcomes and was, therefore, removed from further analysis [69].

## 7. KIM-1 in Myocardial Infarction 

Progressive decline in renal function coexists with myocardial infarction (MI), although mechanisms underlying its dysfunction are poorly understood. The mortality of these patients is high (it is assumed that 20% of hospitalized patients with acute MI have renal impairment and around 25% of them die during hospitalization) [70–72]. The pathogenesis may include an inflammatory response after MI and various cytokines, such as IL-6, TNF-*α*, IL-1*β*, and transforming growth factor-*β* (TGF-*β*), which appear to be major contributors to renal fibrosis [73–75].

Additionally, neurohormonal activation and hemodynamic disturbance (mainly the renin-angiotensin-aldosterone and sympathetic nervous system activation) that have been demonstrated in both humans and animals after acute MI may affect cardiac pump function leading to systemic hypotension and hypoperfusion of all organs, including kidney [76–79].

In a recent study, conducted by Lekawanvijit and colleagues in a rat MI model, they examined potential mechanisms of development of renal changes by monitoring time-course renal functional, structural, and molecular changes following acute MI. They showed kidney injury molecule-1-positive staining in the tubules of experimental animals just one week after MI and concluded that KIM-1 may be a potentially useful kidney injury biomarker for early detection and monitoring of disease progression [80].

## 8. KIM-1 in Organ Transplantation

Acute graft dysfunction provoked by immunological or ischemic injury leads to severe obstacles. In this sense, finding markers that could predict potential organ donors, early posttransplant periods, and long-term follow-up represent a crucial step in further studies [81].

Another potential application of, for example, kidney-injury-specific biomarkers is for guiding decisions on when to initiate renal replacement therapy (RRT). It is well known that KIM-1 values increase in acute graft rejection, but its role in delayed graft function (DGF) is still obscure [82].

Recently, one small study linked urinary KIM-1 as a positive predictor of 14-week and 1-year posttransplantation serum creatinine. KIM-1 values were measured in tissue and urine in 20 brain death kidney donors before organ removal and these were compared with living donors before nephrectomy. Tissue KIM-1 mRNA was 2.5-fold and urinary KIM-1 was twofold higher in brain death donors when compared with living donors [83].

First prospective study that has examined the relationship of preimplantation tissue KIM-1 expression with immediate and long-term graft function was conducted by Schröppel et al. They measured KIM-1 RNA and protein expression in preperfusion biopsies of 30 living-donor and 85 deceased-donor kidneys and correlated the results with histologic and clinical outcomes after transplantation. Their results showed that tubular KIM-1 expression correlated with eGFR at the time of organ procurement but did not correlate with the incidence of DGF [84].

A recent study has explored urinary biomarkers in 63 renal transplant recipients who showed decline of renal function. Urinary KIM-1 expression is marked as significant predictor of prognosis in these patients (group with high KIM-1 expression has significantly worse graft survival) [85].

Besides that, it is important to point out that KIM-1 is not the most useful biomarker in prediction of DGF. Peake et al. showed in their study that urinary levels of KIM-1 increased after transplantation peaking at 24 h and remained higher than those in control subjects 168 h after transplantation but did not correlate with early graft outcome [86]. Recently published paper by Pianta et al. showed that clusterin and IL-18 are more useful markers in triaging of patients with DGF within 4 h of transplantation [87].

## 9. KIM-1 in Critically Ill Patients 

Acute kidney injury is one of the most frequent problems occurring in the critically ill patients in the intensive care units. Despite novel therapeutic strategies, it remains an unresolved problem in pharmacotherapy with high mortality rate, incidence of which varies from 28% to 90% [88, 89].

Despite this, until now, just few studies have examined the importance of the application of new biomarkers in these patients. One of them evaluated its interest in critically ill patients and indicated KIM-1 as a potential marker for prediction of the need for RRT and 7-day mortality [90].

Another study described that urinary KIM-1 levels correlated with dialysis requirement and hospital mortality in 201 critically ill hospitalized patients who developed AKI. A more recent report in the pediatric literature described a 252-patient cohort study in which KIM-1 levels predict the development of AKI in the emergency department [66].

Regardless, it is true that KIM-1 has not proved particularly effective in predicting clinical outcome in critically ill patients in some other studies. For example, Endre et al. conducted prospective observational study among patients in general intensive care units in order to better understand the diagnostic and predictive performance of some urinary biomarkers of kidney injury. Comparisons were made using the area under the curve (AUC) for diagnosis or prediction of acute kidney injury (AKI), dialysis, or death. It was shown that KIM-1 was not particularly useful in prediction of dialysis and death in 7 days, although its utility was improved with stratification for duration of AKI and baseline GFR [91].

## 10. Limitation of KIM-1 as a Diagnostic and Prognostic Marker in Cardiovascular Diseases

Previous research indicates the importance of the introduction of KIM-1 as a diagnostic and prognostic marker in kidney and heart disease. Nevertheless, it should be noted that it is unlikely that a determination of the one single marker may be sufficient in many clinical entities with very complex pathogenesis and diverse etiology. For such complicated processes it is more appropriate to combine biomarkers to maximize the features and to minimize disadvantages of each one [19].

At this point it seems that KIM-1 represents a promising candidate for inclusion in the urinary “AKI Biomarker Panel” together with NGAL. One advantage of KIM-1 as a urinary biomarker is the fact that its expression seems to be limited to the injured or diseased kidney, although its value may affect number of other confounding variables [20]. KIM-1 in the kidney and urine is also induced in a variety of chronic proteinuric, inflammatory, and fibrotic disease states in humans [92].

According to Endre and Pickering, the need for biomarker panel may include the requirement for heterogeneity in timing (biomarkers have varying time courses which are usually shorter than that of creatinine), heterogeneity of etiology (biomarker levels are dependent on preexisting conditions, and some of them may influence biomarker threshold), and heterogeneity of background function (e.g., reduced baseline glomerular filtration rate also modifies the concentrations and time course of both injury and function biomarkers) [93]. The advantages and disadvantages of KIM-1 biomarker are presented in Table 1.

## 11. Conclusion

Numerous animal and human studies promoted KIM-1 as a promising, new biomarker for early diagnosis, monitoring of therapeutic effects, and prediction of clinical outcome in cardiovascular diseases. Also, it should be pointed out that precise assessments of validity and establishing standards for measurement of KIM-1 as a novel marker in preclinical and clinical studies are highly required. At this moment, larger trials are necessary before a strong endorsement for establishment of KIM-1 in broader clinical use.

Finally, we believe that the future studies will demonstrate the right place and the right role of each of novel biomarkers in clinical use, including KIM-1, which was evaluated in this paper.

## Figures and Tables

**Table 1:** Advantages and disadvantages of KIM-1 (adapted from [19, 20]).

Advantages	Disadvantages
Can detect AKI earlier than serum creatinine	Primarily research tools

May suggest type of acute kidney injury	Is itself enough in diagnosis and prognosis, just as a part of “panel of biomarkers”

Can be measured in tissue, urine, and serum/plasma	Can be affected by numerous confounding variables

Urinary kidney injury molecule-1 (KIM-1) is a marker of tubular damage	Needs validation in appropriate clinical settings

Good sensitivity and specificity	High cost and poor availability

High prognostic value	

ELISA commercial assay	
